# Comparison of prevalence and exposure-disease associations using self-report and hospitalization data among enrollees of the world trade center health registry

**DOI:** 10.1186/s12874-021-01358-y

**Published:** 2021-08-10

**Authors:** Howard E. Alper, Jennifer Brite, James E. Cone, Robert M. Brackbill

**Affiliations:** New York City Department of Health and Mental Hygiene, World Trade Center Health Registry, 30-30 47th Avenue, Room 414, Long Island City, NY 11101 USA

**Keywords:** World trade center, 9/11, Disaster, Self-report, Hospitalization, Chronic disease

## Abstract

**Background:**

Although many studies have investigated agreement between survey and hospitalization data for disease prevalence, it is unknown whether exposure-chronic disease associations vary based on data collection method. We investigated agreement between self-report and administrative data for the following: 1) disease prevalence, and 2) the accuracy of self-reported hospitalization in the last 12 months, and 3) the association of seven chronic diseases (rheumatoid arthritis, hypertension, heart attack, stroke, asthma, diabetes, hyperlipidemia) with four measures of 9/11 exposure.

**Methods:**

Enrollees of the World Trade Center Health Registry who resided in New York State were included (*N* = 18,206). Hospitalization data for chronic diseases were obtained from the New York State Planning and Research Cooperative System (SPARCS). Prevalence for each disease and concordance measures (kappa, sensitivity, specificity, positive agreement, and negative agreement) were calculated. In addition, the associations of the seven chronic diseases with the four measures of exposure were evaluated using logistic regression.

**Results:**

Self-report disease prevalence ranged from moderately high (40.5% for hyperlipidemia) to low (3.8% for heart attack). Self-report prevalence was at least twice that obtained from administrative data for all seven chronic diseases. Kappa ranged from 0.35 (stroke) to 0.04 (rheumatoid arthritis). Self-reported hospitalizations within the last 12 months showed little overlap with actual hospitalization data. Agreement for exposure-disease associations was good over the twenty-eight exposure-disease pairs studied.

**Conclusions:**

Agreement was good for exposure-disease associations, modest for disease prevalence, and poor for self-reported hospitalizations. Neither self-report nor administrative data can be treated as the “gold standard.” Which source to use depends on the availability and context of data, and the disease under study.

**Supplementary Information:**

The online version contains supplementary material available at 10.1186/s12874-021-01358-y.

## Background

Effectively monitoring population health for chronic diseases requires reliable and accurate data. The most common type of data for measuring prevalence of chronic disease has been surveys, in which subjects self-report demographic information and chronic disease status. However, surveys require extensive planning and are often expensive to administer. Further, the accuracy of the data obtained can be influenced by sampling and recall bias and other sources of error, themselves influenced by factors such as demographic characteristics and disease status [1–3].

In recent decades, hospitalization data have become more commonly used, due to easier access and increased computational power. Hospitalization data are often perceived as more objective and less biased because they are record-based. Nonetheless, such data have limitations. First, the identifying information contained in administrative data is often limited to portions of the name, date of birth, gender, social security number, and address. This contrasts with the rich demographic, social, and behavioral data often obtained from surveys. Further, coding entry errors can lead to misreporting and under-reporting of chronic disease. Finally, hospitalization data are usually limited to specific geographic regions, and so cannot provide data on subjects outside the coverage region.

Since there are concerns with both survey and administrative data, caution is recommended in accepting either as objective truth. A more prudent approach would be to view the two sources of health data as complementary for the purpose of obtaining a more accurate picture of population health. However, to achieve this end it is important to evaluate the nature and degree of agreement between survey and hospitalization data.

Much research has investigated the concordance between survey self-report and hospitalization data for the prevalence of chronic physical disease. For example, in a regionally representative sample of Quebec citizens [4], the agreement between self-report and administrative data was found to be almost perfect for diabetes (kappa = 0.82), moderate for heart disease (kappa = 0.54), and fair for chronic obstructive pulmonary disorder (COPD) (kappa = 0.23). Similar results were obtained from a study in Ontario [5]. Research in the United States comparing the self-report-based vs. Medicare-based prevalence for heart attack and diabetes found, for both diseases, that while the time trends were similar for both data sources, the prevalence was somewhat higher for self-report than for Medicare-based data [6]. A recent study of 202 Iranian participants in their RaNCD cohort study, measuring agreement between self-report and official medical records by the kappa statistic, found a wide range of agreement for different diseases. Specifically, they found kappa = 0.39 for Hepatitis B and hypertension, kappa = 0.65 for thyroid disease, kappa = 0.87 for ischemic heart disease, and kappa = 1.00 for cancer [7].

Monitoring the health of populations or other groups can also involve investigating the association between relevant exposures and chronic disease outcomes based on subjective self-reported vs. objective sources. However, less work has been done on this aspect of concordance research. One study [8] treated heart attack based on both subjective self-report and administrative data as an exposure, and mortality as the outcome, and investigated their association. They found that among the 3.1% of respondents with self-reported heart attack, 32.8% had a claims-identified heart attack, and among 1.4% of respondants with an administrative record of heart attack, 67.8% reported a heart attack. Self-report and administrative data had similar associations with mortality (Odds ratio = 2.5 vs. 2.8). However, to the best of our knowledge, no concordance study has investigated the association between an environmental exposure and chronic physical disease as the outcome. Disaster research provides an opportunity to explore such exposure-disease associations, since during a disaster a set of subjects experience an exposure, to varying degrees, and some of these subjects subsequently develop one or more chronic physical diseases.

This study employs self-report data collected by the World Trade Center Health Registry, as well as New York State hospital discharge data matched to World Trade Center Health Registry enrollees, to evaluate the concordance between these two sources for defining physical health outcomes. The aims of this study are: 1) ascertain whether prevalence of chronic physical disease is similar in self-report compared to hospitalization data for the period 2002–2015, 2) quantify accuracy of self-report of a hospitalization within the last 12 months, 3) determine whether the association between exposure to the 9/11 attacks and physical chronic disease is similar when evaluated using self-report and hospitalization data sources.

## Methods

### Setting

The World Trade Center Health Registry was established in 2003 to monitor the physical and mental health consequences of the terrorist attacks of September 11th, 2001. Enrollees included rescue/recovery workers, residents, area workers, passersby, and students/staff of local schools.

### Data collection

The initial World Trade Center Health Registry survey was conducted in 2003–2004 (wave 1), and subsequently in 2006–2007 (wave 2), 2011–2012 (wave 3), and 2015–2016 (wave 4). Survey data were collected via mail, web, and computer aided telephone interview (CATI). The methods used by the World Trade Center Health Registry have been described in detail in previous publications [9, 10]. The World Trade Center Health Registry was approved by the institutional review boards of the Centers for Disease Control and Prevention and the New York City Department of Health and Mental Hygiene.

### Administrative data

Hospital discharge data were obtained from the New York State Department of Health’s Statewide Planning and Research Cooperative System (SPARCS). SPARCS contains data on all inpatient and emergency department discharges in New York State, except for federal and psychiatric hospitals. Each SPARCS discharge record contains, in addition to personal identifier data (e.g. date of birth), an admitting diagnosis, the principal diagnosis and 24 secondary diagnoses. For the period under investigation, 2002–2015, the SPARCS system typically contains 2–2.5 million records per year.

A World Trade Center Health Registry-SPARCS matched dataset was created by matching records from the two sources, based on a deterministic algorithm using parts of the first and last names, as well as date of birth, sex, social security number, and zip code in hierarchical rounds. SPARCS data covering the period 2002–2015 were employed in the match. Both inpatient and emergency department visits were included. This matched dataset included World Trade Center Health Registry enrollees with hospitalizations in the above period, approximately 60% (*N* = 42,292) of the total World Trade Center Health Registry cohort (*N* = 71,426).

### Analytic sample

**Fig. 1:**
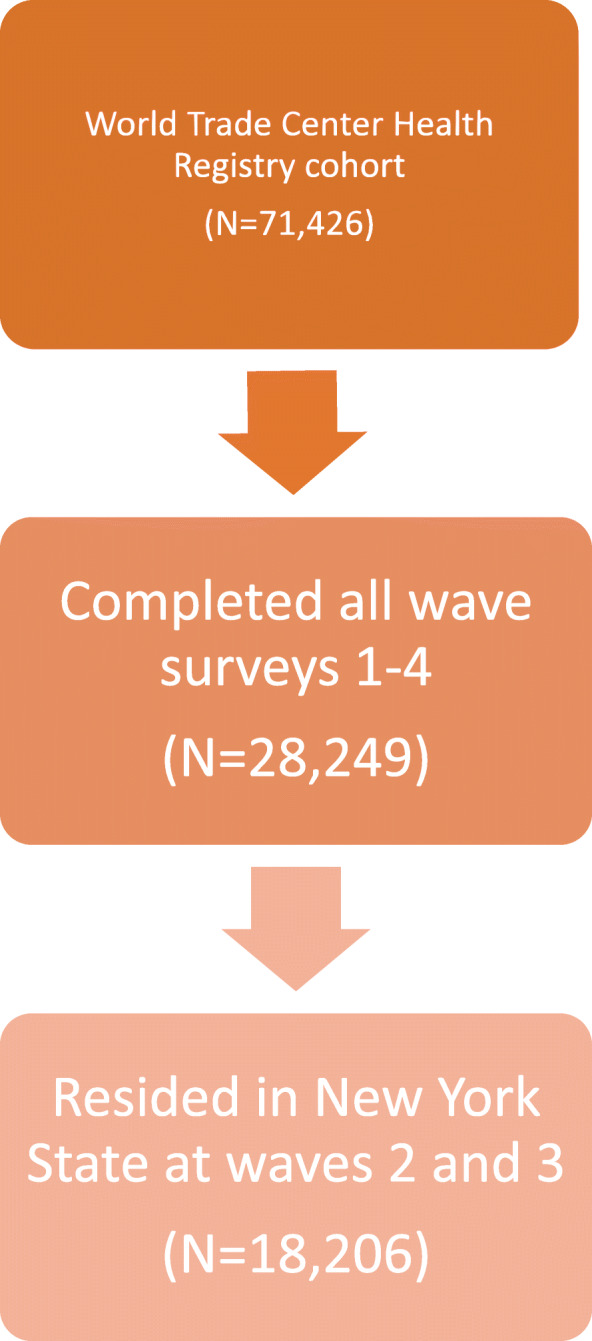
Development of the study analytic sample

The analytic dataset was obtained from the full World Trade Center Health Registry cohort (*N* = 71,426) using the following inclusion criteria: 1) Enrollees must have completed all four survey waves (*N* = 28,249); 2) enrollees must have resided in New York State at waves 2 and 3 (*N* = 46,028; see Fig. 1). The final analytic dataset (*n* = 18,206) included both self-report and SPARCS data.

### Outcome measures

Seven chronic diseases were investigated: Rheumatoid Arthritis, hypertension, heart attack, stroke, asthma, diabetes, and hyperlipidemia (elevated cholesterol). Two binary (yes/no) outcomes were created for each disease: self-report (SR) and hospitalization (SPARCS).

Self-report binary outcome for the period 2002–2015 was obtained from the following question in the waves 2–4 World Trade Center Health Registry surveys: “Have you ever been told by a doctor or other health professional that you had any of these conditions? If yes, please provide the year you were first told you had that condition”. If the enrollee reported having the disease at any of these waves, with a year of diagnosis between 2002 and 2015, the self-report outcome was defined as yes. If the enrollee reported having the disease at waves 2–4, but the year of diagnosis was never between 2002 and 2015 for any of these waves, the self-report outcome was defined as no. If the enrollee did not report having the disease at any of waves 2–4, the self-report outcome was defined as no.

The administrative binary outcome for the period 2002–2015 was set to yes if any of the enrollee’s hospitalization(s) in the SPARCS-World Trade Center Health Registry dataset contained International Classification of Diseases (ICD-9) codes for the relevant disease, in either the SPARCS principal diagnosis or the twenty-four secondary diagnoses. Otherwise, the binary outcome was set to no for that disease. ICD-9 codes relevant to the seven diseases studied here are given in the Appendix 1.

In addition to analysis for the period 2002–2015, we compared self-report and hospitalization data for the shorter period preceding wave 3, using the self-reported question “During the last twelve months, have you been hospitalized overnight for this condition?” from the wave 3 survey. Both self-report and hospitalization outcomes were defined as binary. If the enrollee self-reported a hospitalization for the disease within 12 months prior to wave 3, the self-reported binary outcome was defined as yes. If the enrollee had a SPARCS hospitalization within 15 months prior to have 3, to account for telescoping in self-reported hospitalization, the hospitalization outcome was defined as yes. If the enrollee self-reported or had a SPARCS hospitalization for the disease outside of twelve or fifteen, respectively, months prior to wave 3, or if there was no self-report or SPARCS hospitalization for the disease, the relevant binary outcome was defined as no.

### Exposure variables

Four variables were selected as exposures for analysis of exposure-disease associations: 1) Post-Traumatic Stress Disorder (hereafter PTSD) (yes/no), assessed at wave 1, 2) caught in the dust cloud (yes/no) on 9/11, 3) injured on 9/11 (yes – 1 or more injuries/no – zero injuries), and 4) witnessed three or more traumatic events on 9/11 (yes/no).

Post-Traumatic Stress Disorder (yes/no) (PTSD) was assessed with the PTSD Checklist-Specific (PCL-S), a 17-item self-reported symptom scale that specifically targets the events of September 11. Probable PTSD was defined as a PCL score ≥ 44. The PCL scale possesses good psychometric properties [11, 12].

The dust cloud exposure was defined as binary (yes/no), based on the question “Was subject outdoors within dust cloud on 9/11/01” from the wave 1 survey. Being injured on 9/11 was derived from a wave 1 survey variable stating the number of injuries (excluding eye injuries) sustained on 9/11 and was treated as binary (yes – 1 or more injuries/no – zero injuries). Witnessing three or more traumatic events (e.g. seeing planes hits buildings, witnessing people jumping from buildings) was also defied as binary (yes – 3 or more events/no – 0, 1, or 2 events), and was obtained by summing questions from the wave 1 survey events enrollees reported having witnessed.

### Covariates

Covariates of interest included gender (male, female), race (White non-Hispanic, Black non-Hispanic, Hispanic, Asian, and Other), age at 9/11, and educational attainment (high school/GED or less, some college, and college grad/post-grad).

### Statistical analysis

The main analyses focused on comparison of self-report of disease to hospitalization for the disease, for the period 2002–2015. The wave 3 survey contained, in addition to the disease self-report question, a subsidiary one asking if the enrollee had been hospitalized for that disease in the previous 12 months. We employed both questions to compare the self-reported vs. SPARCS-derived rates of hospitalization within the year preceding wave 3. Since the analytic sample contained emergency department visits, in addition to inpatient visits, we performed a sensitivity analysis by performing the same calculations as above using only inpatient visits. We performed a second sensitivity analysis by performing the above calculations using only the principal diagnosis from inpatient visits.

The disease prevalence, based (separately) on self-report and hospitalization data, was calculated as the ratio of the number of enrollees with the disease to the analytic sample size (*n* = 18,206). Concordance measures were also obtained for each disease between self-report and hospitalization prevalence estimates, that included the kappa statistic, sensitivity (of self-report vs. SPARCS), specificity (of self-report vs. SPARCS), positive agreement, and negative agreement.

We also examined the association of each disease with the four measures of exposure to the 9/11 attacks using both data sources. This was done using logistic regression between each disease outcome-exposure pair. All regressions controlled for age at 9/11, race/ethnicity, gender, and education. Odds ratios (OR): and their 95% confidence intervals (CI) were taken as the measure of association and its statistical significance. We investigated the difference between the self-report and SPARCS odds ratios on the log scale (i.e. the difference between the β) for all twenty-eight exposure-disease pairs and assessed the statistical significance of these differences.

All calculations were performed using SAS 9.4 (Cary, North Carolina).

## Results

The characteristics of the study sample (*n* = 18,206) are summarized in Table 1. 60.5% were male, 69.8% were White non-Hispanic, 51% were aged 25–44 years on 9/11, 52% had a college degree or post-graduate education, and 14.9% had probable PTSD at wave 1.

**Table 1:** Demographic characteristics of the analytic sample

Characteristic	N	%
Gender		
Male	10,910	60.5
Female	7116	39.5
Race		
White non-Hispanic	12,586	69.8
Black non-Hispanic	1858	10.3
Hispanic	2081	11.5
Asian	1011	5.6
Other	490	2.6
Age at 9/11		
0–17	223	1.2
18–24	578	3.2
25–44	9185	51.0
45–64	7625	42.3
65+	414	2.3
Education		
High School Diploma or less	3989	22.2
Some College	4570	25.4
College Degree/Post grad	9431	52.4
PTSD at Wave 1		
Yes	2666	14.9
No	15,201	85.1
Exposed to Dust Cloud		
Yes	10,262	57.2
No	7675	42.8
Injured on 9/11		
Yes	2641	14.8
No	15,261	85.2
Witnessed Traumatic Events on 9/11		
Yes	6823	38.6
No	10,855	61.4

The disease prevalence and concordance statistics are presented in Table 2. For all diseases, self-report prevalence was greater than hospitalization-based prevalence, by a factor of two or greater. Self-report- and hospitalization-based prevalence differences were small for some diseases (e.g., 2.4% vs. 1.1% for stroke), but moderately large for other diseases (e.g. 36.5% vs. 17.9% for hypertension). The kappa ranged from fair (0.35 for stroke) to slight (0.04 for rheumatoid arthritis). For all diseases, sensitivity was less than specificity, and positive agreement less than negative agreement. These latter differences were attenuated for diseases where the prevalence was larger (e.g., hypertension, hyperlipidemia).

**Table 2:** Chronic disease prevalence and agreement statistics

Disease	Prevalence (SR)^a^	Prevalence (SPARCS)	Kappa	Sensitivity	Specificity	Positive Agreement	Negative Agreement
Rheum. Arth.	9.5	0.4	0.04	0.60	0.91	0.05	0.95
Hypertension	36.5	17.9	0.21	0.61	0.69	0.40	0.78
Heart Attack	3.8	1.6	0.31	0.56	0.97	0.33	0.98
Stroke	2.4	1.1	0.35	0.57	0.98	0.36	0.99
Asthma	18.1	6.9	0.23	0.55	0.85	0.30	0.91
Diabetes	12.2	5.8	0.34	0.61	0.91	0.39	0.95
Hyperlipidemia	40.5	11.2	0.10	0.59	0.62	0.26	0.74

^a^SR Self-report

Self-report and SPARCS-derived hospitalizations within 12 months of wave 3 are shown in Table 3. For rheumatoid arthritis, heart attack, and stroke, the number of self-reported hospitalizations exceeded the SPARCS-based hospitalizations. The reverse was observed for hypertension, asthma, and diabetes. However, the overlap between the two data sources was extremely low for all six diseases, kappa statistics were all in the slight range. The results changed little if emergency department visits were excluded, or if only principal diagnoses were included (see Supplements S1 and S2). These results were somewhat smaller than those calculated for the 2002–2015 time period, in Table 2.

**Table 3:** Hospitalization Self-Report at Wave 3 and Verification by SPARCS

Disease	Self-Report Hospitalization at Wave 3	SPARCS Hospitalization Within 15 Months^a^ Before Wave 3	Overlap of Self-Report and SPARCS for Wave 3	Kappa
Rheum. Arth.	39	7	0	0.00
Hypertension	194	322	32	0.11
Heart Attack	79	23	9	0.17
Stroke	50	11	3	0.10
Asthma	94	125	9	0.08
Diabetes	66	80	6	0.08
Hyperlipidemia^b^	–	–	–	–

^a^A 15-month period was employed for the SPARCS data to account for possible telescoping in the self-report data

^b^Wave 3 survey did not include hospitalization question about hyperlipidemia

Disease-exposure associations are shown in Table 4. The difference between odds ratios obtained from self-report and SPARCS was non-significant for twenty-four of twenty-eight exposure-disease pairs. For three exposure-disease pairs the difference in odds ratios was statistically significant but the odds ratios did not differ substantially in size. For a single exposure-disease pair the difference in odds ratios was significantly significant, and the odds ratios differed substantially in size.

**Table 4:** Disease-Exposure Associations^a, b, c,d,e^

	**PTSD**		**Dust Cloud Exposure**	
	**SR**	**SPARCS**	**SR**	**SPARCS**
**Disease**	**OR (95% CI**^**f**^**)**	**OR (95% CI)**	**OR (95% CI)**	**OR (95% CI)**
Rheum. Arth.	2.2 (2.0, 2.5)	2.0 (1.1, 3.4)	1.3 (1.1, 1.4)	1.0 (0.6, 1.7)
Hypertension	1.6 (1.4, 1.7)	1.4 (1.3, 1.6)	1.2 (1.1, 1.3)	1.1 (1.0, 1.2)
Heart Attack	2.3 (1.9, 2.8)	1.7 (1.2, 2.2)	1.1 (1.0, 1.3)	1.1 (0.9, 1.4)
Stroke	1.9 (1.5, 2.4)	1.5 (1.1, 2.2)	1.5 (1.2, 1.8)	1.3 (0.9, 1.7)
Asthma	2.1 (1.9, 2.4)	1.9 (1.6, 2.2)	1.3 (1.2, 1.4)	1.3 (1.2, 1.5)
Diabetes	1.7 (1.5, 1.9)	1.6 (1.4, 1.9)	1.2 (1.1, 1.3)^g^	1.0 (0.9, 1.1)^g^
Hyperlipidemia	1.4 (1.3, 1.6)	1.3 (1.2, 1.5)	1.0 (1.0, 1.1)	1.1 (1.0, 1.2)
	**Injury on 9/11**		**Traumatic Events**	
	**SR**	**SPARCS**	**SR**	**SPARCS**
**Disease**	**OR (95% CI)**	**OR (95% CI)**	**OR (95% CI)**	**OR (95% CI)**
Rheum. Arth.	1.6 (1.4, 1.8)^g^	0.6 (0.3, 1.5)^g^	1.3 (1.1, 1.4)	0.7 (0.4, 1.3)
Hypertension	1.3 (1.1, 1.4)	1.2 (1.1, 1.3)	1.2 (1.1, 1.3)^g^	1.0 (1.0, 1.1)^g^
Heart Attack	1.3 (1.1, 1.6)^g^	1.0 (0.7, 1.4)^g^	1.3 (1.1, 1.5)	1.2 (1.0, 1.6)
Stroke	1.3 (1.0, 1.6)	1.1 (0.7, 1.6)	1.4 (1.1, 1.7)	1.0 (0.8, 1.4)
Asthma	1.8 (1.7, 2.0)	1.6 (1.4, 1.9)	1.2 (1.1, 1.3)	1.3 (1.1, 1.5)
Diabetes	1.3 (1.2, 1.5)	1.1 (0.9, 1.3)	1.2 (1.1, 1.3)	1.1 (0.9, 1.2)
Hyperlipidemia	1.2 (1.1, 1.3)	1.1 (0.9, 1.2)	1.1 (1.0, 1.1)	1.0 (0.9, 1.1)

^a^Logistic regression was used to determine exposure-disease associations

^b^Analytic sample size = 18,206 enrollees for all diseases

^c^SPARCS data used for calculations included both IP and ED visits

^d^Regressions controlled for gender, race, age at 9/11, and education

^e^*SR* Self-report

^f^*CI* Confidence interval

^g^Self-report-SPARCS odds ratio pairs in red are statistically significantly different

## Discussion

The present study evaluated the agreement between self-report and hospitalization data sources for chronic physical diseases, based on disease prevalence and on exposure-disease associations, for subjects exposed to the September 11, 2001 attacks. We found only modest agreement for disease prevalence, but good agreement for the association of the seven chronic diseases with four measures of the 9/11 attacks.

While the level of agreement between self-report and hospitalization data for disease prevalence was modest for the study period 2002–2015, the agreement was poor for hospitalizations within 12 months of wave 3. This could result from limitations of the hospitalization data, or possibly because people were not accurate at reporting hospitalizations within a year.

Although we found modest agreement between survey and hospitalization data for disease prevalence, the associations between exposure to the 9/11 attacks and several disease outcomes were similar using both data sources. This finding is analogous to a study [8] that examined heart disease-mortality associations among Medicare recipients using both claims and self-report data for heart disease. One difference between that study and the present one is that we were able to examine whether associations remained constant when both exposure and outcome were self-reported. The current findings suggest that misreporting of disease status is non-differential for the exposure measures employed, at least among this cohort, as the point estimates obtained via hospitalization data are slightly attenuated but close to those obtained via self-report. This provides evidence that either data source may be sufficient for research on the association of chronic physical disease with environmental (or other) exposures, at least for associations measured on a relative scale. This broader result for exposure-disease associations may not hold for associations measured on an absolute scale (e.g. risk difference).

### Strengths and limitations

A major strength of this study is that it employed a large prospective cohort with substantial self-report data on exposure to the 9/11 attacks, as well as on subsequent chronic disease. This allowed us to investigate both disease prevalence and its association with various measures of exposure to a disaster.

An important limitation is we linked World Trade Center Health Registry data to a single source of hospitalization data – SPARCS. We therefore could not obtain data on chronic diseases for enrollees residing outside of New York State or for enrollees in psychiatric institutions. This likely led to reduced prevalence estimates for chronic physical disease from hospitalization data, and to poorer agreement between self-report and administrative data sources.

A further limitation of our study is the attrition that occurs between surveys. Waves 2–4 had response rates of 68, 63, and 55%, respectively. Attrition can potentially introduce bias into the concordance estimates of the present study. However, such bias was found to be small in a previous World Trade Center Health Registry study [13], at least for measures of association.

## Conclusions

This study investigated the concordance between self-report and hospitalization data for the prevalence of chronic physical disease and its association with exposure to the 9/11 attacks. Exposure-chronic disease associations were found to be similar for the two data sources, so either can be used for this purpose. Agreement was less satisfactory for disease prevalence. Choosing a data source therefore should be based on a variety of factors such as availability and research aims.

## Supplementary Information

**Additional file 1: Appendix 1.** ICD-9 Codes for Chronic Diseases.

## Data Availability

The datasets generated and analyzed during the current study are available from the corresponding author on reasonable request.

## Abbreviations

WTCHR: World Trade Center Health Registry
SPARCS: **(**New York) State Planning and Research Cooperative System
SR: Self-Report
PTSD: Post-Traumatic Stress Disorder
OR: Odds Ratio
CI: Confidence Interval
ICD: International Classification of Disease
