# Supplementary material for: Comparison of prevalence and exposure-disease associations using self-report and hospitalization data among enrollees of the world trade center health registry
Source: BMC Med Res Methodol. 2021 Aug 10;21:162. doi: 10.1186/s12874-021-01358-y (PMC8353750; doi:10.1186/s12874-021-01358-y)
Supplement: Supplementary file 1 — Additional file 1: Appendix 1. ICD-9 Codes for Chronic Diseases. [file 12874_2021_1358_MOESM1_ESM.zip › Appendix 02 18 2021 BMC MRM.docx]

Comparison of Prevalence and Exposure-Disease Associations Using Self-Report and Hospitalization Data Among Enrollees of the World Trade Center Health Registry

Howard E. Alper, PhD, MS^1,*^, Jennifer Brite, DrPH^1^, James E. Cone, MD, MPH^1^, Robert M. Brackbill, PhD, MPH^1^

**Appendix: ICD-9 Codes for Chronic Diseases**

| **Chronic Disease** | **ICD-9 Code(s)** |
| --- | --- |
| Rheumatoid Arthritis | 714.XX |
| Hypertension | 401.XX |
| Heart Attack | 410.XX, 428.XX |
| Stroke | 430.XX, 431.XX, 432.XX, 435.XX, 436.XX, 433.01, 433.10, 433.11, 433.21, 433.31, 433.91, 434.00, 434.01, 434.11, 434.91 |
| Asthma | 493.XX |
| Diabetes | 250.XX |
| Hyperlipidemia | 272.0X, 272.1X, 272.2X, 272.3X, 272.4X |
